# Direct contact and environmental contaminations are responsible for HEV transmission in pigs

**DOI:** 10.1186/1297-9716-44-102

**Published:** 2013-10-28

**Authors:** Mathieu Andraud, Marine Dumarest, Roland Cariolet, Bouchra Aylaj, Elodie Barnaud, Florent Eono, Nicole Pavio, Nicolas Rose

**Affiliations:** 1 Anses, Laboratoire de Ploufragan/Plouzané, Unité Epidémiologie et Bien-Être du Porc, BP 53, 22440 Ploufragan, France; 2 Université Européenne de Bretagne, 5 Boulevard Laennec, 35000 Rennes, France; 3 UMR 1161 Virologie Anses/INRA/ENVA, laboratoire de Santé Animale, 23 Avenue du General de Gaulle, 94706 Maisons-Alfort, France; 4 Département de Mathématiques et informatique, Faculté des sciences Aïn chock, Université Hassan II, Maarif, B.P 5366, Casablanca 20100, Maroc

## Abstract

Hepatitis E virus (HEV) can cause enterically-transmitted hepatitis in humans. The zoonotic nature of Hepatitis E infections has been established in industrialized areas and domestic pigs are considered as the main reservoir. The dynamics of transmission in pig herds therefore needs to be understood to reduce the prevalence of viremic pigs at slaughter and prevent contaminated pig products from entering the food chain. An experimental trial was carried out to study the main characteristics of HEV transmission between orally inoculated pigs and naïve animals. A mathematical model was used to investigate three transmission routes, namely direct contact between pigs and two environmental components to represent within-and between-group oro-fecal transmission. A large inter-individual variability was observed in response to infection with an average latent period lasting 6.9 days (5.8; 7.9) in inoculated animals and an average infectious period of 9.7 days (8.2; 11.2). Our results show that direct transmission alone, with a partial reproduction number of 1.41 (0.21; 3.02), can be considered as a factor of persistence of infection within a population. However, the quantity of virus present in the environment was found to play an essential role in the transmission process strongly influencing the probability of infection with a within pen transmission rate estimated to 2 ⋅ 10^− 6^*g ge*^− 1^*d*^− 1^(1 ⋅ 10^− 7^; 7 ⋅ 10^− 6^). Between-pen environmental transmission occurred to a lesser extent (transmission rate: 7 ⋅ 10^− 8^*g ge*^− 1^*d*^− 1^(5 ⋅ 10^− 9^; 3 ⋅ 10^− 7^) but could further generate a within-group process. The combination of these transmission routes could explain the persistence and high prevalence of HEV in pig populations.

## Introduction

Hepatitis E virus (HEV) is a small non-enveloped single-stranded virus, sole representative of the *Hepeviridae* family [1-3]. It is the causative agent of hepatitis E in humans and transmitted by the oro-fecal route. In most cases, the disease is self-limiting with clinical features similar to hepatitis A and generally does not progress to chronicity, except in immunocompromised humans [4,5]. However, 1 to 2% of cases can experience more severe forms of the disease leading to hepatic failures [6]. Four HEV genotypes have been described to date and circulate in different geographic areas. Genotypes 1 and 2 are exclusively human viruses mainly prevalent in developing countries in Asia, Africa and Central-America [7-9]. In these areas, hepatitis E occurs both sporadically and in epidemic waves, due to poor sanitation and drinking-water contamination. A different situation is observed in industrialized countries where sporadic HEV cases are reported. Although some of these cases can be linked with travel history in developing areas, the number of diagnosed autochthonous cases has increased dramatically in the last decade [10]. These locally acquired infections are commonly associated with genotypes 3 and 4 [9] which are shared between humans and animals, and are especially prevalent in domestic and wild pigs. Although the origin of most autochthonous HEV cases remains undetermined, some cases have been related to the consumption of uncooked meat, liver or offal from domestic pigs or wild animals (e.g. wild boars or deer) [11-13]. Moreover, some studies reported sequence identities close to 100% between human and local swine HEV strains and effective cross-species transmission was experimentally shown for genotypes 3 and 4 [14,15]. Hepatitis E is thus recognized as an emerging zoonosis for which domestic pigs are considered as the main reservoir [3,10,16].

Hepatitis E virus is widespread in domestic pig populations and several studies have shown high prevalence at the herd level with large variations at the individual pig level [17-20]. A recent epidemiological study carried out in France revealed seroprevalences of 65% and 31% at farm- (*n* = 186) and individual-levels (*n* = 6565 blood samples), respectively [19]. The broad distribution of within-herd seroprevalences observed in this study suggests that the spread of infection within the pig population was influenced by farm-dependent characteristics. As in humans, transmission of hepatitis E virus between pigs is assumed to occur mainly by the oro-fecal route [21]. Although pigs of all ages have been found to shed virus in their feces, peak prevalence is generally observed in three to four month-old pigs and in only a small proportion of six month-old animals [18,22,23]. Thus HEV infections occur in young animals (3 months old on average) with further propagation within and between pig groups. In mathematical models, the transmission intensity is governed by the transmission rate, denoted *β* and defined as the average number of new infections produced by one typical infected pig per time unit. This parameter is a key component of epidemiological models, along with the mean duration of the infectious period. These parameters are required to define the basic reproduction number (*R*_0_), which is defined as the average number of new infections produced by one infectious individual during its entire infectious period in a fully susceptible population. Satou and Nishiura [24] developed a model that accounted for the distribution of time between infection and seroconversion to determine the age at infection, and estimated the basic reproduction ratio (*R*_0_ = 4.02 – 5.17) from serological data in pig-farms in Japan. Recently, Backer et al. [25] obtained similar *R*_0_ values when they used a Bayesian framework to analyze the prevalence of HEV shedding according to age group, in pig farms in the UK. In contrast, Bouwknegt et al. [26], using data from experimental transmission trials, obtained higher *R*_0_ values. This experiment was based on serial 1 to 1 transmission experiments and intravenous inoculation of the initial seeder pig. Furthermore, Bouwknegt et al. [27] developed a dose–response model to study the contribution of feces as a source for HEV transmission among pigs. However, the dose–response relationship was obtained after intravenous inoculation with subsequent extrapolation to oro-fecal infection, assuming that oro-fecal contamination was 10^4^ times less effective [28]. The authors showed that the oro-fecal route of infection was likely, but not sufficient to explain the observed transmission, and concluded that other transmission routes needed to be considered to explain the dynamics of infection. Although these previous results provide new insights regarding HEV transmission patterns they are difficult to extend to a real pig farm population that consists of small groups of animals with an extremely heterogeneous contact structure. Moreover, the persistence and accumulation of Hepatitis E viruses in the environment due to fecal shedding could be an important factor for viral transmission among pigs and has not been investigated to date.

Considering the high prevalence of HEV in pig herds and the zoonotic nature of the infection, an in-depth understanding of within-herd HEV dynamics should facilitate the identification of suitable measures to decrease HEV transmission between animals. The aim of the present study was therefore to analyze the main characteristics of HEV infection dynamics in pig populations, based on an experimental transmission trial involving orally inoculated pigs and specific-pathogen-free contact pigs. First, the distributions of the durations of latency and infectious periods were determined. Although infection from a contaminated environment is considered as the primary route of infection, close contact due to coprophagous behavior in pigs might be considered as a non-negligible source of transmission via direct contact between pen-mates. Thus, transmission parameters were estimated considering (i) direct transmission between pen-mates, (ii) within-pen environmental transmission, and (iii) between-pen environmental transmission representing the transfer of fecal material from pen to pen.

## Material and methods

### Data

#### Inoculum

Viral suspensions were prepared from the liver of a pig experimentally infected with hepatitis E virus (genotype 3, Genebank accession number JQ953665), homogenized in PBS (pH 7.2) and centrifuged at 1000 *g* at 4 °C for 20 min. The resulting clear supernatant was purified by passage through microfilters with a pore size of 0.45 μm (Millex-GV; Millipore SAS, Molsheim, France). The amount of HEV was determined by real-time quantitative RT-PCR (qRT-PCR) as described below.

#### Animals, housing conditions and preliminary study of HEV infectivity per os

All experiments were conducted in our air-filtered level 3 biosecurity-facilities. Piglets were obtained from the Anses SPF (specific pathogen free) pig herd and were 6 weeks old at the beginning of the experiments. This herd is HEV-free and the piglets do not have any maternal antibodies. The piglets were housed in metallic flat decks with a punched floor for feces and urine evacuation. As in the field situation, fecal material could accumulate in the corners, and was not removed during the trial.

A preliminary study was conducted to determine the infectious dose resulting in successful pig infection after oral inoculation as well as the potential transmission of HEV from inoculated to naive animals. Twenty-four piglets were assigned to five groups: one control group (four pigs) and four other groups of five animals. Three pigs in each of these four groups were orally inoculated with 10 mL of a viral suspension titrating 10^4^, 10^5^, 10^6^ or 10^8^ genome equivalents per mL, respectively. The suspension was administered using a soft sterile catheter that was gently but deeply introduced into the oesophagus to prevent the inoculum from flowing back and further contaminating the environment. All pigs were monitored for 26 days to detect the success rate of the inoculations as well as potential transmission of the virus to susceptible animals.

#### Transmission experiment design

**Figure 1:**
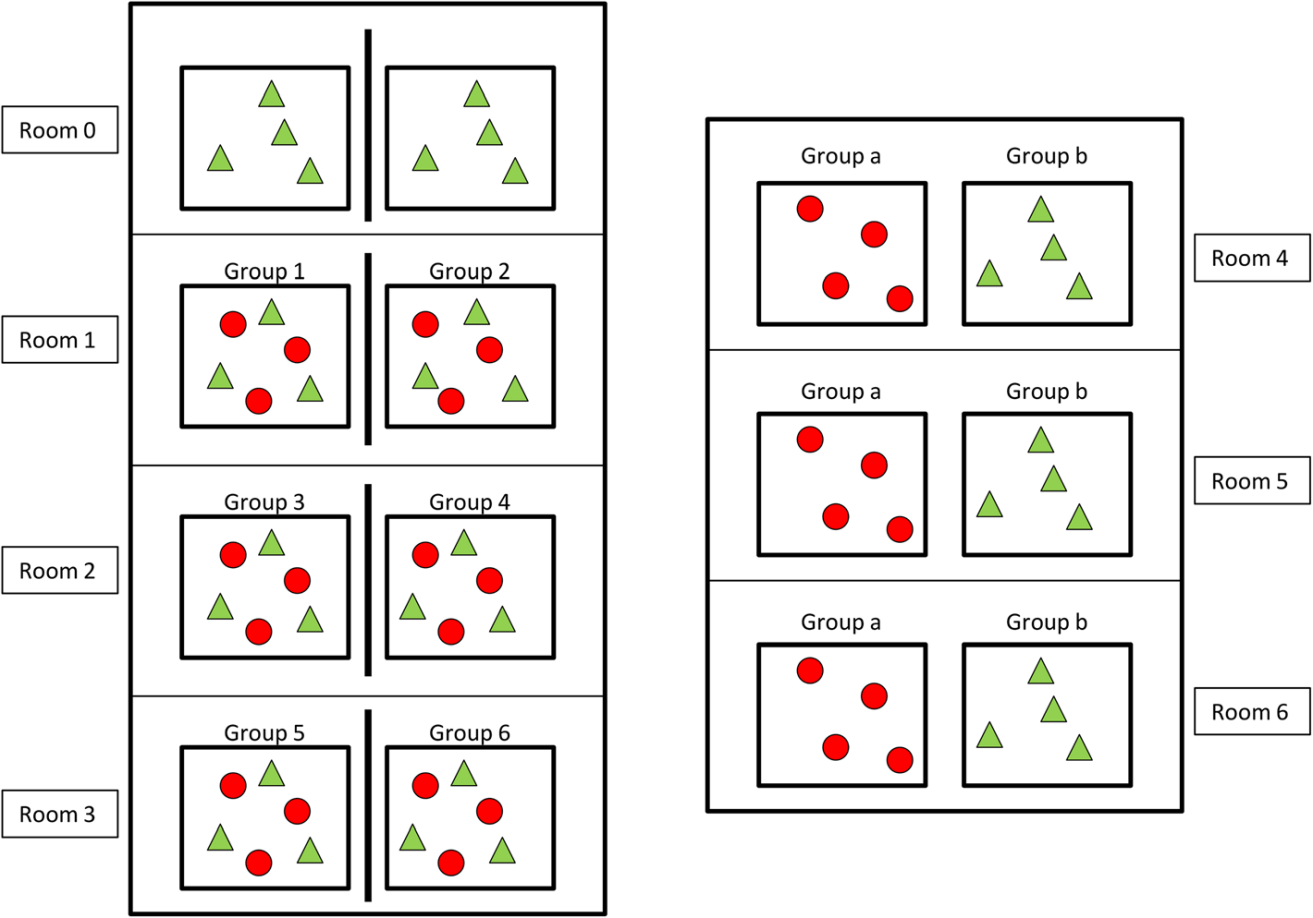
**Experimental design of the transmission experiment.** Inoculated and susceptible animals are represented by red circles and green triangles, respectively. Rooms 1 to 3 contained two pens housing 3 inoculated (red circles) and three susceptible contact pigs (green triangles) and were used to assess transmission by direct contact between animals as well as transmission via the environment. Rooms 4 to 6 were dedicated to the study of the transmission between animals in two adjacent pens separated by 10 cm. One negative control group was housed in room 0.

Sixty-eight piglets were used for the transmission experiment. Eight pigs were kept as negative controls (room 0) and the others were allotted to six rooms containing two pens per room (Figure 1). Rooms 1 to 3 were used to evaluate direct and environmental transmission. The pens in these rooms were separated by solid partitions and housed six randomly selected pigs, three of which were orally inoculated at day 0 (D0). Rooms 4 to 6 were used to examine between-pen transmission. Two adjacent pens, 10 cm apart, housed four inoculated and four SPF contact pigs respectively (Figure 1). A viral suspension titrating 10^8^ ge/mL was selected for the inoculations, based on the results of the pre-infectivity study. Pigs received 10 mL of this viral suspension by oral route. The experiment was performed in accordance with EU and French regulations on animal welfare in experimentation. The protocol was approved (opinion 14/06/2011-3) by the Anses/ENVA/UPEC ethical committee (agreement #16 with the National committee for Ethics in animal experimentation).

#### Sample collection

Individual fecal samples were collected four days before inoculation and three times per week from 0 to 39 days post-infection (dpi) when the pigs were killed for necropsy. Blood samples were collected twice a week during the same period, and clinical signs and rectal temperatures were monitored on a daily basis. Euthanasia was carried out by anaesthesia by intravenous injection of 1 g/50 kg liveweight of Nesdonal® (Merial, Lyon, France) followed by exsanguination.

Since the hepatitis E virus presents zoonotic risks, strict biosecurity measures were implemented to prevent possible transmission from the pigs to the animal technicians. Gloves and facial masks were used systematically. Specific equipment was used for each experimental room. In each pen, samples were taken first from SPF contact pigs and then from inoculated animals. In addition, waders and boots were thoroughly cleaned and disinfected between each pen to prevent transfer of fecal material from one pen to another.

#### Analyses

HEV RNA quantification was adapted from the method described by Johtikumar et al. [29] and performed as described in Barnaud et al. [30]. Briefly, TaqMan RT-PCR was performed using the QuantiTec Probe RT-PCR Kit (Qiagen, Courtaboeuf, France) according to the manufacturer’s instructions with 2 μL of RNA (template). Reverse primer (5′-AGGGGTTGGTTGGATGAA-3′) was used at a final concentration of 0.25 μM, forward primer (5′-GGTGGTTTCTGGGGTGAC-3′) at 0.1 μM and probe (FAM-TGATTCTCAGCCCTTCGC-MGB) at a final concentration of 5 μM. A Light Cycler apparatus (Roche Molecular Biochemicals, Meylan, France) was used for sample analysis. Reverse transcription was carried out at 50 °C for 20 min, followed by denaturation at 95 °C for 15 min. DNA was amplified immediately with 45 cycles at 95 °C for 10 s and 58 °C for 45 s. The final extension was followed by cooling at 40 °C for 30 s. Real-time RT-PCR data were collected after the reaction and the crossing points (CP) were calculated. Standard quantification curves were produced by plotting the CP values against the logarithm of the input copy numbers of standard RNA. Standard RNA was obtained after in vitro transcription of a plasmid pCDNA 3.1 ORF2-3 HEV, as described in Barnaud et al*.*[30,31].

### Models

#### Latency and infectious periods

The distributions of the durations of the latent and infectious periods for HEV infection in pigs were examined. For each animal, the first positive fecal sample for HEV RNA was considered as the beginning of the infectious period and corresponded to the latency duration in inoculated animals. Thus, data from inoculated animals was used to determine the distribution of the latent period. The pigs were then considered infectious until the last positive fecal sample. Gamma-distributions were assumed for both durations, from which the shape and rate parameters were estimated by maximum log-likelihood method. A nonparametric bootstrap procedure was used to determine the 95% confidence intervals of the parameter estimates. One thousand datasets were generated by resampling the original dataset. For each bootstrap replicate, the model was refitted to get the parameter estimates. The 95% confidence interval was constructed from the 2.5^th^ and 97.5^th^ percentiles for each parameter [32,33].

#### Environmental viral load

The environmental viral load corresponds to the accumulation of viral particles in the environment through fecal shedding by infected animals, which is partially compensated by a clearance rate, hereafter denoted *δ*. For each pen (*k*) and every sampling time (*t*_*i*_), the average quantity of genome equivalent shed in the environment per gram of feces was calculated by $V_{k}\left(t_{i}\right)=\Sigma_{j}\;V_{k}^{j}\left(t_{i}\right)/N_{k}$, where $V_{k}^{j}\left(t_{i}\right)$ represents the quantity of virus shed per gram of feces in pen *κ* by pig *j* at time *t*_*i*_$(V_{k}^{j}\left(t_{i}\right)=0$ for a non shedding animal *j* at time *t*_*i*_), and *N*_*k*_ the total number of animals in pen. Thus the cumulated viral load in the environment of pen *k* between two sampling times *t*_*i*_ and *t*_*i+*1_ is governed by the equation:

$$E_{\mathrm{ki}}=E_{k}\left(t_{i+1}\right)=\left(E_{k}\left(t_{i}\right)+\int_{0}^{\mathrm{\Delta t}}V_{k}\left(t_{i}+u\right)e^{\mathrm{\delta u}}\mathrm{du}\right)e^{-\mathrm{\delta \Delta t}},$$

with Δ*t = t*_*i+*1_ - *t*_*i*_. In this relation, the time points before the first positive sample were neglected because they would have induced an unknown but similar dilution factor in each pen for the first positive environmental load, without any dramatic impact on the global evolution of the viral loads in the environment. The quantity of virus shed in the environment by fecal route between two sampling times was approximated by linear interpolation.

#### Quantification of transmission

Three transmission routes were investigated in this study: (i) transmission due to direct contact between infected and naïve pigs; (ii) oro-fecal transmission via an environmental reservoir of virus as described in the previous section; (iii) environmental transmission to pigs housed in adjacent pens. A Bayesian framework was used to estimate the transmission parameters [34,35], considering the latency period and the viral clearance in the environment as random variables. Hence, the shape and rate parameters of the Gamma distribution representing the duration of latency period in naturally infected animals were also estimated, the probability density function being further denoted *f*_*Lat*_(*t*, *A*, *B*). On each sampling interval *D*_*i*_ = [*t*_*i*_, *t*_*i* + 1_] of duration *d*_*i*_, the probability for a susceptible pig *j* housed in pen *k* to escape infection is given by [36,37]:

$$p_{i}^{\left(k\right)}=\exp\left(-d_{i}\left(\beta_{w}\pi_{i}^{\left(k\right)}+\beta_{E}^{\left(w\right)}\;\frac{E_{\mathrm{ki}}^{\left(w\right)}}{N}+\beta_{E}^{\left(b\right)}\frac{E_{\mathrm{ki}}^{\left(b\right)}}{N}\right)\right)$$

where $\pi_{i}^{\left(k\right)}$ represents the proportion of shedding pigs in the time interval *D*_*i*_ located in pen *k*. As defined in the previous section, $E_{\mathrm{ki}}^{\left(w\right)}$ and $E_{\mathrm{ki}}^{\left(b\right)}$ represent the environmental pool of viral particles in time interval *D*_*i*_ in pen (within pen) and in the adjacent pen (between pen), respectively. *β*_*w*_ is the within-pen transmission rate by direct contact, $\beta_{E}^{\left(w\right)}$ the within-pen environmental transmission rate and $\beta_{E}^{\left(b\right)}$ the environmental transmission rate exerted by the adjacent pen (*b*etween pen). Let $D_{I_{j}}=\left[t_{I_{j}},t_{I_{j}+1}\right]$ denote the time interval during which the first positive fecal sample was detected in pig *j*. The contribution of contact animal *j* in pen *k* to the likelihood, i.e. the probability for its first positive fecal sample to stand in the interval $D_{I_{j}}=\left[t_{I_{j}}+t_{I_{j}+1}\right]$:

$$\begin{array}{l} L^{\left(j\right)}\left(\left(D_{I},\pi_{w},E\right|\beta_{w},\beta_{E}^{\left(w\right)},\beta_{E}^{\left(b\right)},A,B,\delta \right)\\ \;=\sum_{i=1}^{I_{j}}\left\{\prod_{l=1}^{i}\;p_{l-1}^{\left(k\right)}\left(1-p_{i}^{\left(k\right)}\right)f_{\mathrm{Lat}}\left(t_{I_{j}}-t_{i},A,B\right)\right\} \end{array}$$

and

$$\begin{array}{l} L\left(\left(D_{I},\pi_{w},E\right|\beta_{w},\beta_{E}^{\left(w\right)},\beta_{E}^{\left(b\right)},A,B,\delta \right)\\ \;=\prod_{j=1}^{N_{c}}\;L^{\left(j\right)}\left(\left(D_{I},\pi_{w},E\right|\beta_{w},\beta_{E}^{\left(w\right)},\beta_{E}^{\left(b\right)},A,B,\delta \right) \end{array}$$

where *N*_*c*_ is the total number of contact pigs.

Transmission parameters, parameters governing the distribution of the latency period in naturally infected pigs and the clearance rate of virus in the environment (*δ*) were estimated by Bayesian inference using Monte Carlo Markov Chain. Vague uninformative uniform prior distributions were used for the three transmission parameters, whereas informative gamma priors based on the estimated parameters of the latency distribution in inoculated animals were used for A and B (Γ(13.48,0.41) and Γ(15.55,0.05), respectively). A mildly informative prior distribution, based on expert opinion, was used for the environmental clearance rate, which was assumed to be normally distributed with mean 0.3 and standard deviation 0.075 (95% CI: 0.15; 0.45). Although the decay rate for HEV is relatively low, the range of clearance rate parameter, between 0.15 and 0.45 per day, permits to account for variability in persistence of the virus in the environment due to the loss of fecal material through flat decks.

Parameter updating was performed sequentially by Metropolis-Hastings algorithm with gamma proposal distributions for all parameters. Three chains were run with random initial conditions, 10000 steps per chain, a burnin of 1000 steps and thinning parameter of 10. Convergence was assessed by visual inspection and diagnostic tests (autocorrelation, Heidelberger, Gelman-Rubin diagnostics). Two nested model structures were tested to analyze whether the direct contact transmission plays a role in transmission. The first model considered environmental transmission alone by forcing *β*_*w*_ to 0 and estimating the four remaining parameters; the second model being the full model in a 5-parameter space. Model selection was based on Deviance Information Criterion.

## Results

### Preliminary study of HEV infectivity per os

All pigs inoculated with viral suspensions titrating 10^4^ and 10^5^ ge/mL remained negative for HEV RNA in feces (Table 1). All but one inoculated animals were successfully infected with the inoculum titrating 10^6^ ge/mL. Moreover, one contact pig in this group shed the virus in its feces. Finally, with inoculum titrating 10^8^ ge/mL, all inoculated and contact animals were found, at least once, to be positive for HEV RNA in fecal samples between 9 and 19 days post inoculation, indicating a high success rate for the inoculations and effective transmission to naïve piglets. In view of these preliminary results, an inoculum titrating 10^8^ ge/mL was used in the transmission experiment. The inoculated and contact pigs in the different groups did not exhibit any clinical signs and rectal temperatures remained normal until the end of the experiment. No macroscopic lesion could be observed at necropsy except an enlargement of the hepatic lymph nodes in several piglets.

**Table 1:** Results of the preliminary infectivity study after oral HEV inoculation in pigs.

**Inoculum titer (ge/mL)**	Number of infected pigs* (Total number of pigs)	
	**Inoculated (*n*** **= 3)**	**Contact (*n*** **= 2)**
10^4^	0	0
10^5^	0	0
10^6^	2	1
10^8^	3	2

*The number of infected pigs corresponds to the number of pigs with at least one HEV positive fecal sample during the 26-day experiment.

### Infection data and estimation of durations of latency and infectiousness

**Figure 2:**
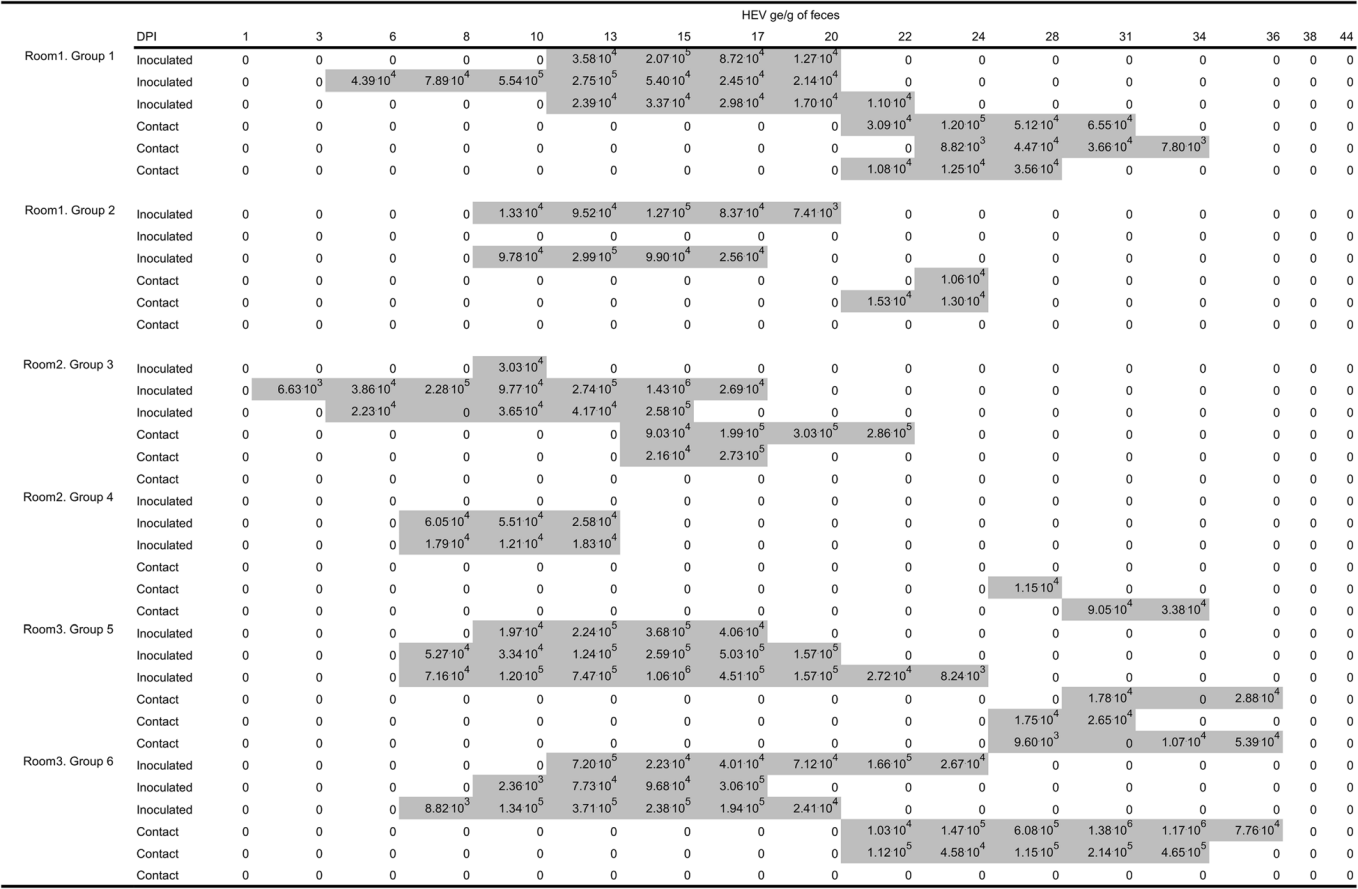
**Quantitative RT-PCR results on individual fecal samples in direct contact groups (rooms 1 to 3).** Shaded zones correspond to periods on which infected individuals were considered infectious, corresponding to the time between the first and last HEV positive fecal samples for each animal.

**Figure 3:**
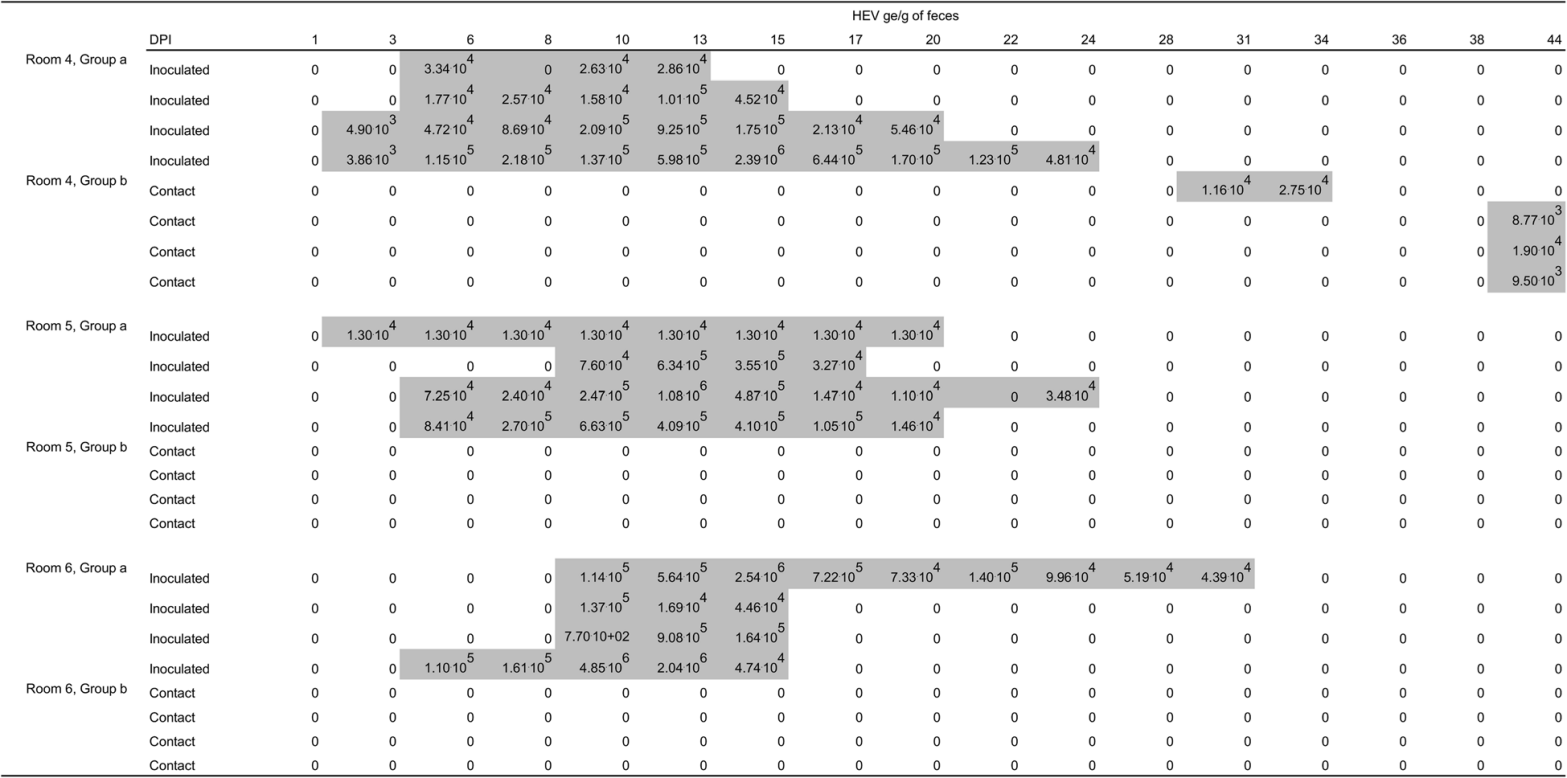
**Quantitative RT-PCR results on individual fecal samples in indirect contact groups (rooms 4 to 6).** Shaded zones correspond to periods on which infected individuals were considered infectious, corresponding to the time between the first and last HEV positive fecal samples for each animal.

**Figure 4:**
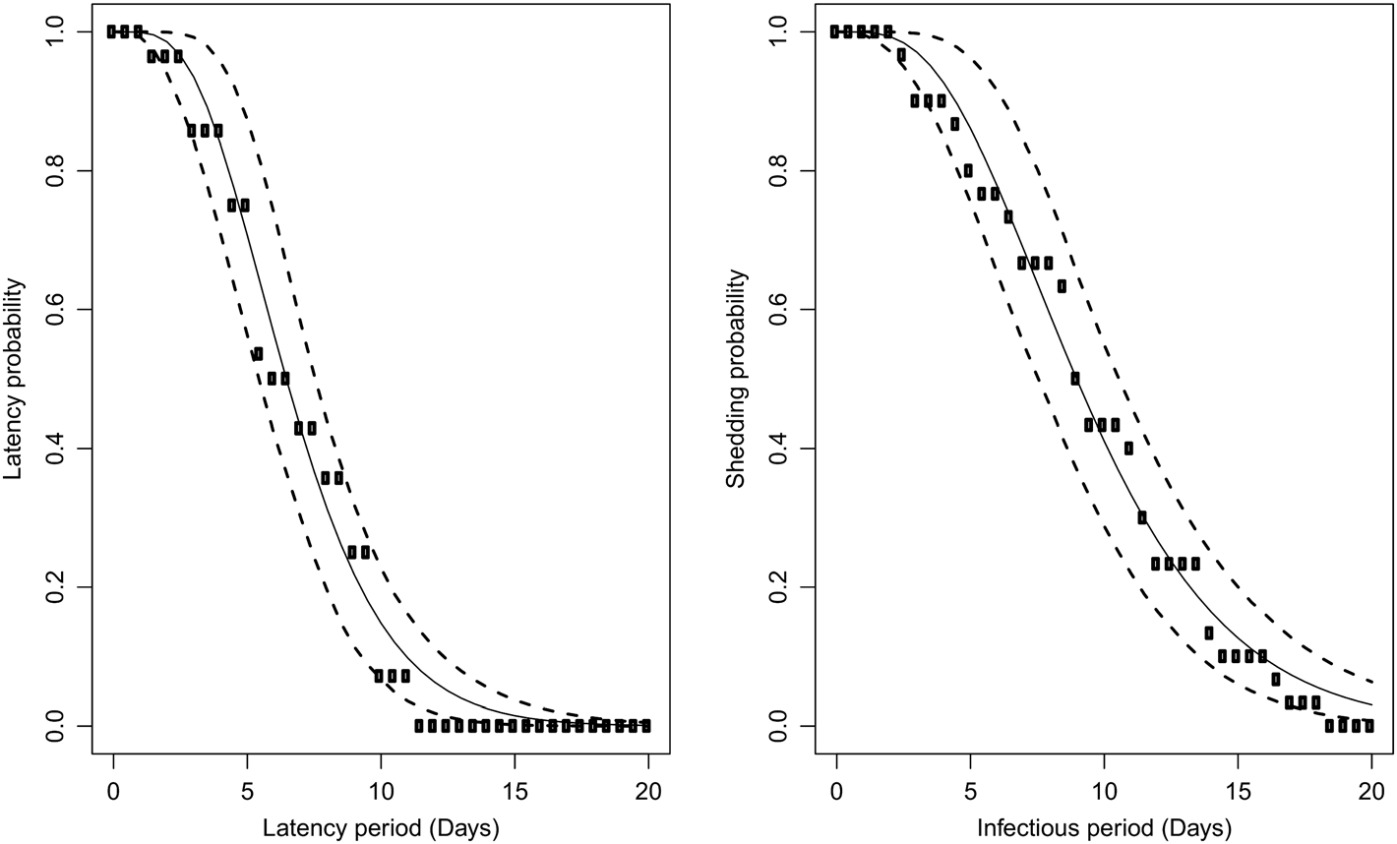
**Fits of the distribution of the latent (left panel) and infectious (right panel) periods.** We assumed gamma distributions for both periods and used the maximum likelihood method to estimate the shape and scale parameters (full lines). 95% confidence bands (dashed lines) were obtained by the bootstrapping method (see text for details).

**Figure 5:**
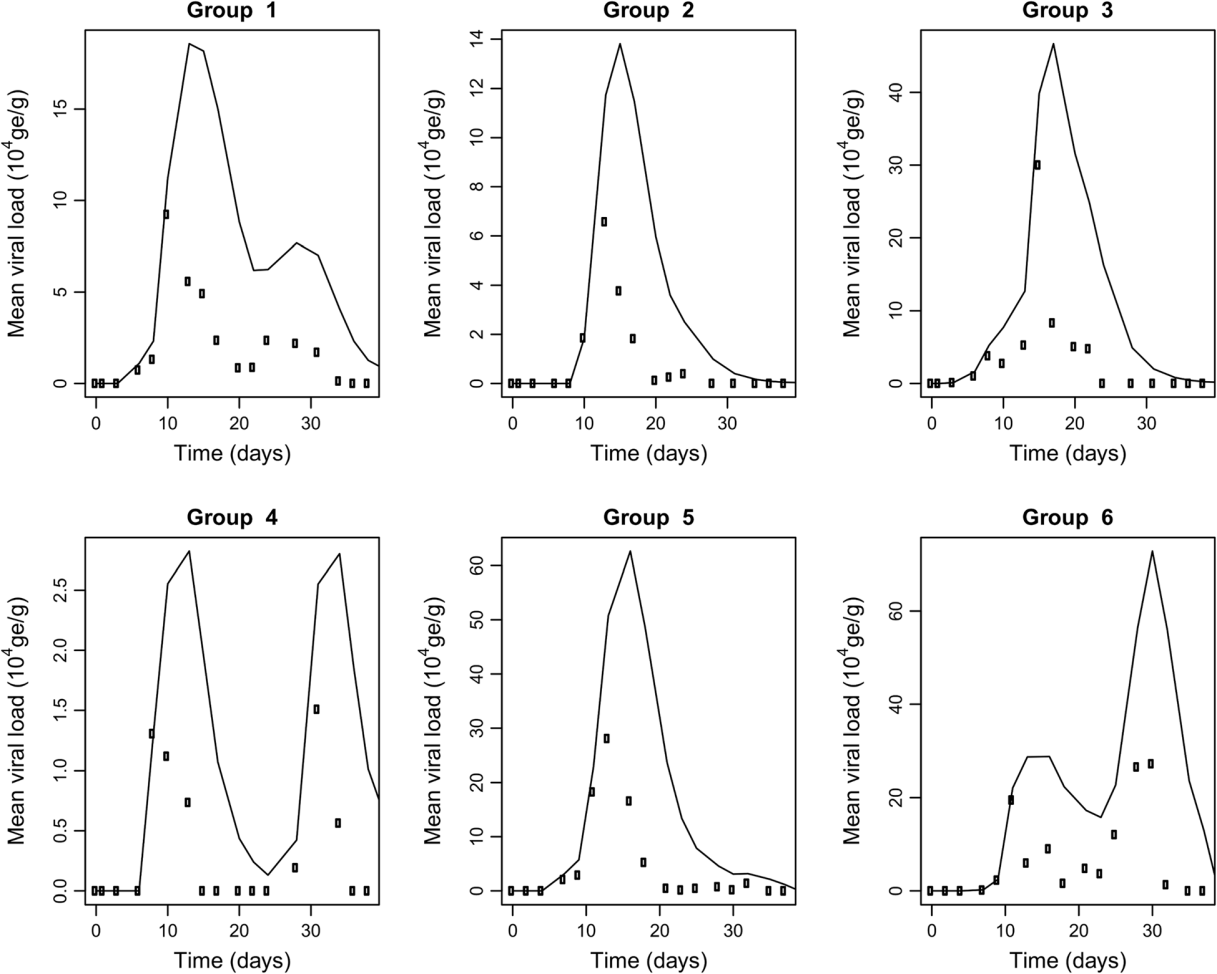
**Estimated environment viral loads in groups 1 to 6.** The average quantity of genome equivalent per gram of feces shed in the environment was first evaluated (dots) at each sampling-time. The environmental viral load (full lines) corresponds to the accumulation of viral particles in the environment through fecal shedding by infected animals, which is partially compensated by a clearance rate.

Inoculations were administered orally so as to be as close as possible to infections in field conditions. Infection was successful in all but two inoculated animals for which all fecal samples were negative for HEV RNA (Figure 2 and Figure 3). Moreover, these two pigs were seronegative at the end of the trial (data not shown), showing that they were not infected by contact with infectious animals and/or material during the entire experimental period. These two animals were considered as susceptible in the estimation process. Inoculated pigs exhibited latent periods ranging from 2 to 11.5 days. The durations of the latent period after inoculation were fitted to a gamma distribution with shape parameter *a*_*L*_ = 5.2 (3.5; 10.0) and rate parameter *γ*_*L*_ = 0.75 (0.52; 1.39) (Figure 4, left panel), leading to an estimated mean duration of the latent period of 6.9 days (5.8; 7.9). Using the same methodology and based on data from inoculated and contact animals, the estimated mean duration of the infectious period was 9.7 days (8.2; 11.3) (Figure 4, right panel). All successfully inoculated pigs were found to shed viral RNA in their feces according to a unimodal curve but with large inter-individual variability. Viral shedding peaks occurred between 8 and 17 days post-inoculation. Although most animals shed less than 5⋅10^5^ ge/g of feces during the entire experiment, two of them in groups 3 and 5 shed more than 10^6^ ge/g and thus contributed to a rapid increase of the viral load present in the environment (Figure 5).

Fourteen of the eighteen naïve pigs in direct contact with inoculated animals (groups 1–6), were infected during the experiment (Figure 2). The first positive fecal sample in contact pigs was observed at 15 dpi in group 3 in which the inoculated animals were infectious soon after inoculation (2 – 5 dpi) and had the highest amount of viral RNA in their feces. In the other groups, fecal samples from contact pigs were found positive for HEV RNA between 21 and 30 dpi. In group 4, a gap of 14 days was observed between the last positive fecal sample in inoculated pigs and the first positive sample in contact pigs. Thus, in this specific group, direct transmission was unlikely therefore suggesting oro-fecal contamination through the environmental reservoir of the virus. Environmental transmission between adjacent pens was effective in only one transmission experiment (Room 4, group b, Figure 3). Moreover, positive samples were obtained from only one pig in this group, from 31 to 36 dpi, whereas the three remaining contact pigs were positive at 44 dpi, suggesting the occurrence of within-group transmission after the first infection.

### Environmental viral load

The quantity of virus present in the environment was approximated in each group from the quantity of virus shed in feces by infected animals. The profiles of environmental viral load in each group in direct contact are shown in Figure 5 assuming a baseline clearance rate of 30% per day. Groups 2, 3 and 5 exhibited similar single-mode profiles reflecting the high quantities of virus shed mainly by inoculated pigs, as well as a rapid spread of the infection among susceptible pigs. According to individual data, the infected contact animals in groups 1 and 6 exhibited longer shedding periods leading to an increase in the environmental load around 30 dpi. Moreover, the individual RT-PCR results for infected contact pigs in group 6 revealed that these animals shed more than 10^6^ ge/g of feces, thus indicating the essential contribution of these pigs to the environmental viral load. The profile observed in group 4 was totally different with two modes separated by a time-interval of 20 days between each mode. In this group, a lag phase of 14 days was observed between the last positive fecal sample from inoculated pigs and the first positive sample from contact animals. Although the approximated amount of virus present in the environment was relatively low in regard to other groups, oro-fecal transmission probably occurred because of the permanent exposure of contact animals to the contaminated environment.

### Quantification of transmission

Two model structures were compared to test whether direct transmission was necessary to explain data or if environmental transmission could be enough to represent the transmission process (forcing the parameter *β*_*w*_ to 0). Model selection, performed using Deviance Information Criterion (DIC), shows that the full model, including direct transmission, better described the data (*Δ*_*DIC*_ > 5; data not shown). The results reported in Table 2 consist of the median value and 95% credibility interval for each estimated parameter considering the best fitting model structure (full model).

**Table 2:** Parameter estimates by the MCMC method.

		**Parameter estimates**	
**Parameter**	**Definition**	**Median**	**95% credibility interval**
*β*_*w*_(*d*^-1^)	Direct transmission rate	0.15	(0.03; 0.31)
*β*_*E*_^*(w)*^ (*g ge*^-1^*d*^-1^)	Within-pen Environmental transmission	2 · 10^-6^	(1 · 10^-7^; 7 · 10^-6^)
*β*_*E*_^*(b)*^ (*g ge*^-1^*d*^-1^)	Between-pen Environmental transmission	7 · 10^-8^	(5 · 10^-9^; 3 · 10^-7^)
*A*	Shape parameter^§^	5.3	(3.2; 8.7)
*B*	Rate parameter^§^	0.76	(0.48; 1.25)
*δ*(*d*^-1^)	Clearance rate	0.33	(0.19; 0.46)

*β*_*w*_ is the direct transmission rate, defined as the mean number of newly infected pigs generated by a single infectious individual in a fully susceptible population per time unit. $\beta_{E}^{\left(w\right)}$, $\beta_{E}^{\left(b\right)}$ represents the within- and between-pen transmission rates related to the environmental component, defined as the mean number of newly infected pigs per viral particle per gram of feces in the environment (see text for more details).

^§^*A* and *B* are respectively the shape and rate parameters of the gamma distribution assumed for the latency duration in contact animals (probability density function (*f*_*Lat*_ (*t*, *A*, *B*)).

Latency duration in contact animals was found similar as in inoculated animals, with a mean latency period of 7.1 days (95% credibility interval: 3.2; 12.3). The posterior mean clearance rate was estimated at 0.33 per day (95% credibility interval: (0.19; 0.46)). The Bayesian framework developed for estimation of transmission parameters accounted for variability caused by the latency period duration and clearance rate. Our results show that, under experimental conditions, one shedding pig would be able to transmit the virus to 0.15 pig per day by direct contact (*β*_*w*_ = 0.15 (0.03; 0.31)). With an estimated average duration of the infectious period of 9.7 days, the “partial” reproduction number, accounting for direct transmission only, was slightly greater than one $\left(R_{0}^{d}=1.41\;\left(0.21;3.02\right)\right)$. Two transmission parameters in relation with the environment were estimated according to the contact structures between individuals. Within-pen transmission rate related to the environment can be considered as the average number of animals that can be infected by a single genome equivalent present in the pen-environment $\left(\beta_{E}^{\left(w\right)}=2\cdot 10^{-6}\;g\cdot {\mathrm{ge}}^{-1}d^{-1}\left(1\cdot 10^{-7};7\cdot 10^{-6}\right)\right)$. Otherwise stated, the inverse of $\beta_{E}^{\left(w\right)}$ corresponds to the average number of viral particles per gram of feces in the environmental pool required to infect one animal in one day, i.e. 5.62 ⋅ 10^5^ ge/g (1.45 ⋅ 10^5^; 9.34 ⋅ 10^6^). The transmission of HEV contaminated feces between adjacent pens was estimated about 30 times less effective than within pen environmental transmission $\left(\beta_{E}^{\left(b\right)}=7\cdot 10^{-8}\;g\cdot {\mathrm{ge}}^{-1}d^{-1}\left(5\cdot 10^{-9};3\cdot 10^{-7}\right)\right)$.

## Discussion

Several studies of experimental HEV-infections by different inoculation methods, such as intravenous (*iv*) or oral inoculations, have been reported [21,26,27,38-42]. Although oro-fecal transmission is considered the most probable route of infection in field conditions, oral inoculation was shown to be less efficient than *iv* inoculation [21,40]. According to Kasorndorkbua et al. [21], oro-fecal transmission of HEV in pigs would require repeated exposures to high doses of virus. Transmission from inoculated to naïve pigs was demonstrated in several studies regardless of the inoculation route. However, most of these studies considered contact-exposure to intravenously inoculated pigs. Bouwknegt et al. quantified the transmission of HEV using three successive generations of contact-exposure pigs, the first generation being obtained by exposure of naïve pigs to animals inoculated intravenously [26]. Considering both direct and indirect transmissions and a constant latent period of three days (based on observations in *iv-*inoculated animals), they obtained a basic reproduction number of 8.8, which appears rather high compared with estimates based on field data [24,25]. More recently, Casas et al. studied transmission from pigs orally inoculated with a bile suspension to naïve piglets [40]. Although oral inoculation was confirmed to be less efficient than intravenous inoculation with 4 successful infections among 16 inoculated pigs, the results highlighted for the first time the effective transmission of HEV to naïve piglets after oral inoculation. However, this study was not designed to quantify the transmission of HEV.

In the preliminary infectivity study, oral inoculations with viral suspension titers below 10^6^ ge/mL were inefficient. This could explain the success rate obtained by Casas and collaborators (25%) since the titer of the bile suspension used for the inoculations was 2⋅10^5^ ge/mL. In the preliminary study, all inoculations with viral suspensions titrating 10^8^ ge/mL were successful and transmission to naïve pigs was effective. Therefore, in the transmission experiment, 26 SPF pigs were orally inoculated with an inoculum titrating 10^8^ ge/mL and 24 (92%) were successfully infected.

Exponential distributions are widely used in epidemiological models to represent the durations of the different disease stages. Thus constant transition rates are used to model the transitions between the stages, irrespectively of the time spent in one specific stage. Although convenient from a mathematical point of view, this assumption is unrealistic as regards the epidemiology of most infectious agents [43]. Moreover, the inclusion of more realistic distributions, such as gamma distributions, can dramatically modify the behavior of a model and have consequences on eventual control measures [44,45]. However, estimating the distributions of latent and infectious periods is often challenging since the true course of infection is generally unknown. This issue can be addressed by using animal experiments since the time of infection corresponds to the time of inoculation and the course of infection in inoculated animals can subsequently be monitored. Considerable inter-individual variability was observed in the kinetics of infection after inoculation especially regarding the durations of the latent and infectious periods. The time lapse between inoculation and the first positive fecal sample ranged between 2 and 13 days and was fitted to a gamma distribution. The estimated mean latent period in orally inoculated animals was 6.9 and 7.1 days in contact animals compared to 3 days after *iv* inoculations [26]. The durations remained comparable in inoculated and naturally infected animals, supporting the hypothesis that oral inoculation is a good model to mimic the infection route in natural conditions. This clearly indicates that the inoculation route influences the within-host kinetics of infection, the latent period being longer in orally inoculated pigs, supporting the results of Bouwknegt et al. [39] who compared the course of infection in intravenously inoculated and contact animals. In the context of oral inoculations, our results show that the pigs shed virus in their feces for 9.7 days (8.2; 11.2) on average which appears relatively short compared with previous estimates [26] (49 days (17; 141); 13 days (11; 17) in two different blocks).

Three transmission parameters were considered in the present study: the first one relies on transmission due to direct contact between animals; the two others are related to an environmental component in the transmission process according to the contact structure (within- and between-pen transmission). Indeed, in addition to the fact that oro-fecal transmission is probably the natural route for HEV infection, the soundness of an environmental transmission representation was confirmed by the observations derived from the experiment: contact pigs in one group (group 4) were found to be infectious about 14 days after the last positive fecal sample had been obtained from inoculated pigs. It is apparent from the mean duration of the latent period in contact animals (7.1 days (3.2; 12.3)), that these late infections were undoubtedly caused by the ingestion of viral material present in the environment. However, including such a component required to represent the amount of virus in the environment. We therefore used the amounts of viral RNA shed in the feces by each animal to approximate the cumulated viral load present in the environment in each pen and accounting for a clearance rate of the virus in the environment, reflecting both the inactivation rate of the virus and the loss of fecal material through the flat decks. The clearance rate was included as a parameter to be estimated in the Bayesian analysis using a mildly informative prior distribution. The comparison of two model structures, including or not the direct transmission between pigs, evidenced that the combination of direct and environmental transmissions best fitted the observed course of infection during the experiment. By analogy to the definitions of direct transmission rates, within-pen environmental transmission parameter, denoted $\beta_{E}^{\left(w\right)}$, reflects the average number of animals infected per genome equivalent per gram of feces in the environment. However, since this definition is highly abstract, we chose to define the relative environmental transmission rate as the inverse of $\beta_{E}^{\left(w\right)}$, which would correspond to the number of viral RNA required to infect one pig (in the absence of direct transmission) and which, consistently with the results of the preliminary infectivity study, was estimated at 5.62 ⋅ 10^5^ *ge*/*g* (1.45 ⋅ 10^5^; 9.34 ⋅ 10^6^). However, direct transmission plays a major role in the transmission process, with an estimated transmission rate of 0.15 (0.03; 0.31). The estimated partial reproduction number, accounting solely for direct transmission, was $R_{0}^{d}=1.41\;\left(0.21;3.02\right)$. Larger *R*_0_ estimates have been previously published for HEV [24-26] without distinct representation of direct and environmental transmission which confirms our findings related to the major contribution of the environment in the overall transmission process. According to our results, a single infectious individual generates through direct contact on average 1.41 infectious individuals during its entire infectious period in a fully susceptible population, thus permitting persistence of the infection within the population. At the same time the environmental load increases as a result of fecal shedding and leads to an increase in the force of infection applied to susceptible individuals. Although the duration of the infectious period was relatively short compared with previous estimates, the accumulation and persistence of viral particles in the environment is pivotal for the spread of infection. In our experimental set-up, environmental transmission between adjacent pens was found to be a rare event. However, as shown by group b in room 4, such transmission can occur, and further generate a within-group transmission process.

Under the experimental conditions of this study, direct and within-pen environmental transmissions were essential components to explain the transmission process, whereas environmental transmission between-pens remained a rare event. In view of these results, strict segregating of pigs in pens and avoidance of movement between pens, could limit the transmission process. HEV propagation within pig populations is therefore highly dependent on pig management and hygiene procedures. The combination of the three transmission processes could lead to a very diverse intensity of virus propagation between animals, which is consistent with the great variability in seroprevalence observed in slaughter pigs between farms [19]. Given the importance of the environmental reservoir in the transmission process, the strict separation of pigs from this pool of virus should shorten the transmission chain to a large extent. Husbandry practices such as intense mingling at different stages, poor hygienic conditions or floor characteristics favoring the persistence of virus can lead to dramatic waterfall sequences and may explain the high HEV prevalence observed in pig herds in several countries [17,19,20,46].

## Competing interests

The authors declare that they have no competing interests.

## Authors’ contributions

MA developed the mathematical model, analyzed the data, performed the parameter estimation and drafted the manuscript. MD performed the samples analysis. RC supervised the animal experiments. BA participated in the parameters estimation. EB developed and validated the quantitative RT-PCR. FE participated in the animal experiments, monitored and pre-treated the samples. NP supervised the laboratory work and participated in the coordination of the study. NR conceived, coordinated the study and participated in the animal experiments. All co-authors revised the manuscript and approved the final submitted version.
